# Mr. Jesse Mercer Battle

**Published:** 1914-11

**Authors:** 


Battle & Co. announce the death on September 16, of their President, Mr. Jesse Mercer Battle, in St. Louis.

